# Detection of air trapping in chronic obstructive pulmonary disease by low frequency ultrasound

**DOI:** 10.1186/1471-2466-12-8

**Published:** 2012-03-16

**Authors:** Katrin Morenz, Heike Biller, Frank Wolfram, Steffen Leonhadt, Dirk Rüter, Thomas Glaab, Stefan Uhlig, Jens M Hohlfeld

**Affiliations:** 1 Institute for Pharmacology and Toxicology, Medical Faculty, RWTH Aachen University, Wendlingweg 2, 52074 Aachen, Germany; 2 Fraunhofer Institute for Toxicology & Experimental Medicine (ITEM), Department of Clinical Airway Research, Nikolai-Fuchs-Str. 1, 30625 Hannover, Germany; 3 Philips Chair of Medical Information Technology, RWTH Aachen University, Pauwelsstraße 20, 52074 Aachen, Germany; 4 Institute for Measurement and Sensor Technology, Mülheim University of Applied Science, Wiesenstraße 36, 45473 Mülheim an der Ruhr, Germany; 5 Boehringer Ingelheim, Medical Affairs, Respiratory, Binger Straße 173, 55216 Ingelheim, Germany

**Keywords:** Bronchodilation, Pulmonary function test, Diagnosis, GOLD

## Abstract

**Background:**

Spirometry is regarded as the gold standard for the diagnosis of COPD, yet the condition is widely underdiagnosed. Therefore, additional screening methods that are easy to perform and to interpret are needed. Recently, we demonstrated that low frequency ultrasound (LFU) may be helpful for monitoring lung diseases. The objective of this study was to evaluate whether LFU can be used to detect air trapping in COPD. In addition, we evaluated the ability of LFU to detect the effects of short-acting bronchodilator medication.

**Methods:**

Seventeen patients with COPD and 9 healthy subjects were examined by body plethysmography and LFU. Ultrasound frequencies ranging from 1 to 40 kHz were transmitted to the sternum and received at the back during inspiration and expiration. The high pass frequency was determined from the inspiratory and the expiratory signals and their difference termed ΔF. Measurements were repeated after inhalation of salbutamol.

**Results:**

We found significant differences in ΔF between COPD subjects and healthy subjects. These differences were already significant at GOLD stage 1 and increased with the severity of COPD. Sensitivity for detection of GOLD stage 1 was 83% and for GOLD stages worse than 1 it was 91%. Bronchodilator effects could not be detected reliably.

**Conclusions:**

We conclude that low frequency ultrasound is cost-effective, easy to perform and suitable for detecting air trapping. It might be useful in screening for COPD.

**Trial Registration:**

ClinicalTrials.gov: NCT01080924

## Background

Chronic obstructive pulmonary disease (COPD) poses a challenge to current and future health care systems. As a result of increased tobacco consumption and demographic development, COPD is expected to become the third leading cause of death worldwide by the year 2020 [1]. Early diagnosis and intervention is necessary to prevent a further decline of lung function in these patients. The Global Initiative for Chronic Obstructive Lung Disease (GOLD) recommends spirometry as the gold standard for the diagnosis of COPD, since it is the most reproducible, standardized and objective way of measuring airflow limitation [2]. However, to perform spirometry, experienced and regularly trained medical assistants are needed as well as physicians for interpreting the results. Possibly due to these problems, spirometry is not frequently used by general practitioners and underdiagnosis of COPD is widespread [3-8]. Therefore, an additional screening method that is easier to perform and to interpret is needed.

Conventional ultrasound with frequencies ranging from 2 to 10 MHz is increasingly used for the diagnosis of pulmonary diseases including pneumothorax, pleural effusion, alveolar-interstitial syndrome and lung consolidation. However, its application is restricted to superficial examination and to abnormally dense lungs [9-12]. The healthy lung cannot be visualized, because differences in acoustic impedances between aerated lung tissue and pleural cavity cause total internal reflection [13].

A novel approach to non-invasive monitoring of the lungs is low frequency ultrasound spectroscopy [14]. Earlier, Goncharoff *et al. *described the sound transmission between 5 and 20 kHz from the mouth to the back [15]. More recently, Rüter *et al. *applying frequencies between 5 and 40 kHz to the sternum demonstrated that the signals received at the back differed between inspiration and expiration in healthy human subjects. The sound spectra changed dependent on the lung aeration: higher aeration resulted in a weaker signal and in a shift of the high pass filter towards higher frequencies. In contrast, in COPD patients the sound spectra during inspiration and expiration remained unchanged [14]. During expiration the signal of COPD patients was similar to the inspiratory signal of healthy subjects, suggesting that this method may be useful for detecting air trapping. In that study, the area under the curve of the sound spectra between 10 and 20 kHz correlated with the forced expiratory volume in 1 sec (FEV_1_) and the intrathoracic gas volume (ITGV) and differed significantly between COPD and non-COPD subjects [14].

Air trapping is a critical clinical feature of COPD. The objective of our study was to evaluate whether air trapping in COPD patients can be detected reliably using low frequency ultrasound (LFU). COPD patients were classified into severity stages GOLD 1-3 [2] and were examined by both body plethysmography and low frequency ultrasound. The amplitude and the high pass frequencies of the sound spectra were compared between COPD patients and healthy subjects. Furthermore we analyzed the bronchodilator effect of salbutamol by body plethysmography and frequency content.

## Methods

### Study subjects

Male or female subjects aged 18 to 70 years with a body mass index ≤ 30 kg/m^2 ^were eligible for the study. Subjects with COPD had obstructive ventilatory dysfunction and the typical symptoms of COPD according to the GOLD guidelines [2]. Healthy subjects were included in the study if they were nonsmokers with normal results in spirometry (FEV_1 _> 80% of predicted and FEV_1_/FVC ≥ 70%) and no history of respiratory disease. Subjects with respiratory tract infection within four weeks before screening and subjects with medical conditions which prohibit the use of salbutamol were excluded. The Hannover Medical School institutional review board approved the study and all patients gave written informed consent.

### Protocol

In a prospective cohort study patients with COPD and healthy subjects were examined with low frequency ultrasound before and after inhalation of salbutamol. After written informed consent had been obtained, the eligibility of subjects was assessed. Eligible subjects were invited for the main study visit. On that day, body plethysmography and low frequency ultrasound were performed simultaneously before and 10-15 min after inhalation of salbutamol. Bronchodilator use followed the recommendations of the ATS/ERS task force [16]. Subjects with COPD inhaled 400 μg salbutamol while healthy subjects received 200 μg salbutamol.

### Low frequency ultrasound

**Figure 1:**
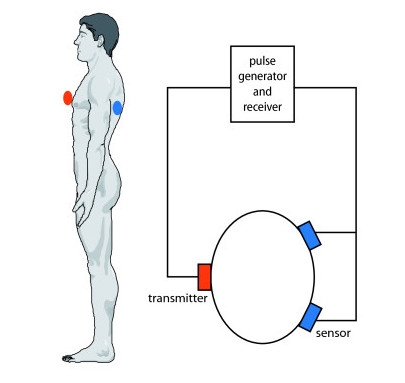
**Measurement setup**. Ultrasound pulses were generated and transmitted to the sternum. After being received at the back by two sensors, the signal was amplified and digitalized.

The measurement setup is shown in Figure 1: A transmitter with a diameter of 35 mm was attached to the lower third of the sternum using an elastic thoracic belt. Two piezoelectric receivers with a diameter of 40 mm were placed on the back at the same height; the use of gel was not necessary. Pulses were generated by a multifunction data acquisition device NI USB-6251 (National Instruments, Austin, TX, USA). The transmitted ultrasound pulses covered a spectrum from 1-40 kHz with a transmit voltage amplitude of ± 15 V and a repetition frequency of 800 Hz. The received signal was amplified and sampled at 125 kHz, resulting in a 16 BIT digital signal. In order to transform the original time function into the frequency domain, a Fourier transform was applied. Matlab (The MathWorks, Natick, MA, USA) was used to control the device and to evaluate the data.

Ultrasound measurements and lung function testing were performed simultaneously. First the ultrasound signal was checked and the sensor position was corrected if necessary. We made sure that we received an adequate signal before beginning the measurements. After starting the lung function testing, the ultrasound signals were saved at the point of maximum inspiration, calm maximum expiration and forced maximum expiration. Ten minutes after inhaling salbutamol the measurements were repeated.

**Figure 2:**
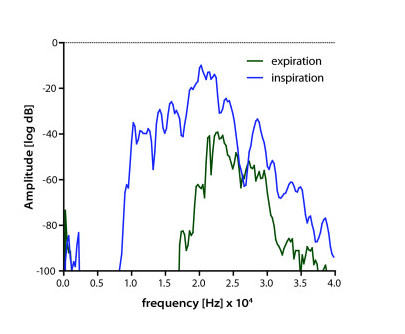
**Frequency spectra at inspiration and expiration**. The lowest frequency of the first strong amplitude signal was measured during inspiration and expiration and the difference between these frequencies was termed ΔF.

The difference between inspiratory and expiratory frequency content was evaluated, as shown in Figure 2. The high pass frequency was determined from the inspiratory and the expiratory signal, which corresponds to the *half value *of the maximum receive voltage on a logarithmic scale. Afterwards the difference between the two high pass frequencies was calculated, giving the frequency shift ΔF. We expected ΔF to increase in response to salbutamol-induced bronchodilation. In addition, we identified the amplitude of the expiratory signal.

### Statistics

IBM SPSS Statistics 18 for Windows (SPSS Inc., Chicago, IL, USA) was used for statistical analysis. Because, in a separate group of healthy individuals, we noted a significant correlation between the body mass index (BMI) and the inspiratory and expiratory signals (Additional file 1; Suppl. Figure 1), we used linear regression to adjust the frequency signal of the following measurements to a BMI of 26 kg/m^2^.

One-way analysis of variance (ANOVA) was used to test for differences in lung function, frequency shift ΔF and amplitude among the groups. Dunnett's post-hoc test was used to analyze differences between the healthy control group and the three COPD groups. Equality of variances was tested using Levene's test; homoscedasticity was assumed if p > 0.01. To compare lung function and frequency shift ΔF before and after bronchodilation, a paired t-test was applied. P < 0.05 was considered statistically significant.

## Results

### Study subjects

Table 1 summarizes the characteristics of the study subjects. Nine healthy subjects and 17 COPD patients were included in the study. One patient was excluded because of increased blood pressure at the screening visit; a reserve patient participated in the trial instead. Three patients with COPD were excluded due to technical reasons. We assume that these subjects braced their shoulders and backs intensely during breathing, thus dislodging the sensors. We were able to reproduce their signals by intentionally shifting the sensors while recording. Twenty-six patients completed the study.

**Table 1:** Characteristics of healthy and COPD subjects classified by GOLD stage I-III*

Characteristic	Healthy (n = 9)	GOLD 1 (n = 6)	GOLD 2 (n = 4)	GOLD 3 (n = 7)
age-yr	38.33 ± 9.72	52.83 ± 10.68	60.25 ± 9.50	59.14 ± 5.08
height-m	1.78 ± 0.06	1.78 ± 0.05	1.75 ± 0.09	1.75 ± 0.05
weight-kg	75.89 ± 11.20	81.50 ± 12.60	79.50 ± 14.27	77.29 ± 9.27
BMI-kg/m^2^	23.94 ± 3.07	25.62 ± 3.59	25.73 ± 2.36	25.33 ± 2.50
FEV_1 _- % pred. pre	101.97 ± 9.36	82.55 ± 11.73	60.75 ± 4.40	31.94 ± 3.02
FEV_1_/FVC-% pre	75.99 ± 2.80	56.98 ± 5.89	47.43 ± 7.06	28.78 ± 4.83
R-kPa · s/l	0.18 ± 0.07	0.24 ± 0.07	0.34 ± 0.16	0.76 ± 0.31
ITGV-l	3.38 ± 0.56	4.93 ± 0.49	4.65 ± 0.46	6.23 ± 0.44
ITGV-% pred.	100.68 ± 13.31	139.12 ± 14.87	131.28 ± 16.49	176.70 ± 14.37
RV-l	1.84 ± 0.16	3.24 ± 0.56	3.32 ± 0.41	4.75 ± 0.62
RV-% pred.	96.16 ± 11.37	142.3 ± 15.22	139.15 ± 16.48	201.59 ± 26.75
FEV_1 _- % pred. post	103.76 ± 9.37	89.47 ± 9.77	72.53 ± 3.40	37.54 ± 6.00
FEV_1_/FVC-% post	77.53 ± 2.64	60.45 ± 5.04	52.06 ± 7.54	29.32 ± 6.03

* Plus-minus values are means ± SD. FEV_1 _and FEV_1_/FVC values are listed before (prae) and after (post) application of salbutamol. There were significant differences in age among healthy subjects and the three COPD groups, as assessed with the use of one-way ANOVA and Dunnett's post-hoc test (GOLD 1: p < 0.01, GOLD 2/GOLD3: p < 0.0001). In lung function testing, FEV_1_, FEV_1_/FVC, ITGV and RV were significantly different (p < 0.0001) between healthy and COPD subjects. Airway resistance (R) was significantly different between healthy subjects and GOLD 3 (p < 0.0001).

### Frequency shift ΔF

**Figure 3:**
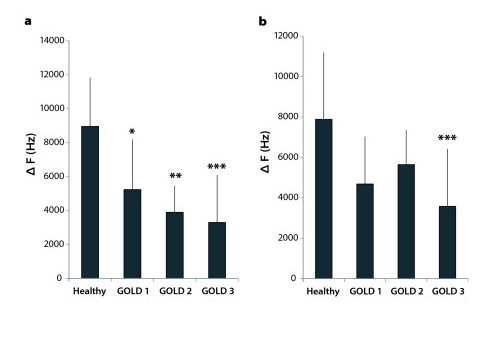
**Frequency shift ΔF at maximum inspiration and expiration**. Frequency shifts ΔF were compared between healthy subjects (n = 9) and COPD subjects classified by GOLD (GOLD 1: n = 6, GOLD 2: n = 4, GOLD 3: n = 7). **a**) During non-forced maximum breathing, one-way ANOVA and Dunnett's post-hoc test showed significant differences between healthy subjects and each GOLD stage. Significance increased from GOLD 1 to GOLD 3 (GOLD 1: p = 0.023, GOLD 2: p = 0.008, GOLD 3: p = 0.0007). **b**) During forced maximum breathing, there was a significant difference between healthy subjects and GOLD stage 3 at p = 0.0008.

The frequency shift ΔF was compared between healthy subjects and COPD patients. The measurements were taken during body plethysmography in non-forced maximum breathing as well as during spirometry in forced maximum breathing. During non-forced maximum breathing, the average frequency shift of healthy subjects was 8958 ± 2874 Hz. In contrast, frequency shifts of COPD subjects were reduced to 58%, 43% and 37% for GOLD 1, GOLD 2 and GOLD 3, respectively (Figure 3). ΔF differed significantly between healthy and COPD patients. The significance increased with the severity of COPD with p = 0.023, p = 0.008 and p = 0.0007 for GOLD 1,2 and 3, respectively.

During forced maximum breathing healthy subjects achieved an average frequency shift of 7882 ± 3310 Hz. As before, the frequency shifts of subjects with COPD were reduced, with values of 59%, 72% and 45% in GOLD 1-3, respectively (Figure 3). During forced maximum breathing, only COPD patients with severe COPD (GOLD stage 3) showed significant differences in ΔF at maximum inspiration and expiration compared to healthy subjects (p = 0.0008).

One aim of this study was to evaluate the sensitivity and specificity of LFU for detecting air trapping in COPD. Therefore, we defined values below the 95% confidence interval from the frequency shift of healthy subjects as being indicative of air trapping (Table 2). As the sample size was small in GOLD stage 2, for this calculation subjects with GOLD 2 and 3 were combined. During non-forced breathing air trapping was recognized in 5 of 6 COPD patients with GOLD stage 1 and in 10 of 11 patients with GOLD stages 2 and 3. Sensitivity for GOLD 1 was 83.3% and 90.9% for GOLD 2/3, the specificity for detection of air trapping of any stage was 88.9%. During forced breathing the LFU method was less effective (Table 2).

**Table 2:** Sensitivity for detection of air trapping

	Sensitivity [%] Non-forced breathing	Sensitivity [%] Forced breathing
GOLD 1	83.3	66.7
GOLD 2 + 3	90.9	72.2

### Amplitude

In healthy individuals, the maximum amplitude from the expiratory frequency spectrum was -35.2 ± 29.1 dB. Values in COPD patients were reduced to -44.8 ± 34.5 dB in GOLD 1, -64.3 ± 30.8 dB in GOLD 2 and -52.7 ± 30.0 dB in GOLD 3. Statistical analysis of the amplitude data in a one-way ANOVA with Dunnett's post-hoc test showed no significant difference from healthy subjects for any of the GOLD stages. Furthermore, the area under the curve was calculated in the interval of 10 to 20 kHz. Values in healthy subjects and GOLD 1 were almost identical, while values in GOLD 2 and GOLD 3 were reduced. In one-way ANOVA with Dunnett's post-hoc test the AUC values from GOLD 3 patients were again significantly different from those of healthy subjects (p = 0.031) [14].

### Bronchodilator response

**Figure 4:**
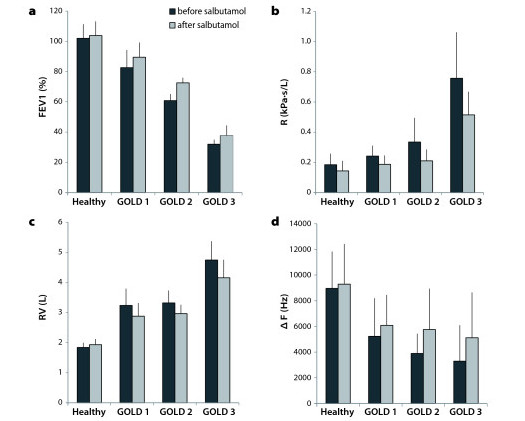
**Lung function and ultrasound before and after inhalation of Salbutamol**. **a**) FEV_1 _increased significantly in healthy and COPD subjects (healthy: p = 0.01, GOLD 1: p = 0.011, GOLD 2: p = 0.015, GOLD 3: p = 0.012). **b**) Airway resistance decreased significantly in healthy subjects (p = 0.0002) as well as in GOLD stage 1 (p = 0.005) and GOLD stage 3 (p = 0.032). **c**) Residual volume decreased significantly in healthy subjects (p = 0.033) as well as in GOLD stage 1 (p = 0.016) and GOLD stage 3 (p = 0.02). **d**) There was no significant difference of frequency shift during non-forced maximum breathing in any group.

After inhalation of salbutamol, the measurements of lung function testing and ultrasound were compared to the original values (Figure 4). The forced expiratory volume in the first second (FEV_1_) increased by 1.75% in healthy subjects, by 8.38% in GOLD stage 1, by 19.38% in GOLD stage 2 and by 17.53% in GOLD stage 3. Airway resistance (R) decreased by 22.3% in healthy subjects, by 22.8% in GOLD stage 1, by 37.3% in GOLD stage 2 and by 32.1% in GOLD stage 3. Residual volume (RV) increased by 5.1% in healthy subjects and decreased by 11% in GOLD stage 1, by 10.7% in GOLD stage 2 and by 12.4% in GOLD stage 3. During non-forced maximum breathing, the frequency shift (ΔF) increased by 4% in healthy subjects. In COPD patients it increased by 16% for GOLD stage 1, by 48% in GOLD stage 2 and by 56% in GOLD stage 3. However, this increase in ΔF in response to salbutamol-induced bronchodilation was not significantly different in any of the groups. During forced maximum breathing the frequency shift increased by 12% in healthy subjects. Subjects with GOLD stage 1 disease had the greatest increase of 46%, while frequency shifts of patients with GOLD stage 2 and 3 disease increased by 17% and 24%, respectively. As in non-forced breathing, these increases were not significant in any group.

### Safety

There were no adverse events reported in this study.

## Discussion

Despite the fact that the physics of low frequency ultrasound are not fully understood, this study shows that air trapping in COPD can be detected by LFU. We found significant differences in ΔF between healthy subjects and COPD subjects during maximum inspiration and expiration. These differences were already significant at GOLD stage 1 and increased with the severity of COPD. The sensitivity for detecting GOLD stage 1 was 83.3%, for detecting moderate to severe COPD (GOLD 2 and 3) it was 90.9%. Our sensitivity analysis (Table 2) showed that these measurements should be obtained during unforced maximum breathing.

Spirometry is required to make the diagnosis of COPD; the presence of a post-bronchodilator FEV_1_/FVC < 0.70 confirms the presence of persistent airflow reduction and thus of COPD according to the GOLD guideline recommendations [2]. Although there is currently insufficient evidence to recommend routine use of low frequency ultrasound, this additional measurement may help to disclose the degree of air trapping in patients with COPD. The method may also be useful in cases where body plethysmography or comparable techniques are unavailable.

In contrast to previous work we found no significant differences in the signal amplitude between COPD patients and healthy subjects [14]. This may be explained by the subject selection, because Rüter *et al. *examined a group of patients with mainly very severe COPD (GOLD stage 4) [14], while we examined COPD patients with GOLD stages 1 to 3. In principle, however, the present study confirms the previous findings [14]. As expected, ΔF increased in response to bronchodilation, but these results were not significant. In contrast, bronchodilation as measured by spirometry and by body plethysmography was associated with a significant increase of FEV_1 _and a significant decrease of airway resistance, respectively. Thus, these conventional measures are more suitable than LFU for assessing bronchodilator responses.

As mentioned above, it is still unclear how low frequency ultrasound is transmitted through the human thorax. Rüter *et al. *showed that with increasing air content of the lungs during inspiration the high pass frequency increased while the signal amplitude decreased. It was suggested that sound traveling through the lungs was influenced by differences in lung density [14]. An important finding in our study was that the typical expiratory signal was received above the abdominal tissue of a healthy person. When placing the sensor above the lower lung border, the signal changed from an expiratory signal with high amplitude and low high-pass frequency during expiration, to an inspiratory signal with lower amplitude and higher high-pass frequency during inspiration. These results can be explained if we assume that the signal is traveling through the abdominal tissue and is attenuated and filtered by the lung, moving into the signal pathway during inspiration. Such a pathway would also explain the dependency on the BMI (Additional file 1; Suppl. Figure 1), because abdominal fat tissue is expected to transmit sound signals well. Thus, the movement of the diaphragm may be the reason for the frequency variability. In COPD hyperinflation of the lungs leads to lowering of the diaphragm and to straightening of the diaphragmatic domes while diaphragmatic movement is reduced [17-19]. Rüter *et al. *described the expiratory signal of a COPD subject as resembling the inspiratory signal of a healthy person [14]. Airway obstruction of COPD patients limits their ability to exhale. This would explain why these patients are not able to achieve the expiratory diaphragmatic position of healthy subjects, resulting in an attenuated signal containing higher frequencies. Accordingly, the frequency variability in COPD patients was less pronounced than in healthy subjects and decreased with air trapping.

At present, the LFU method has a number of limitations that need further clarification. It remains uncertain whether the method can be applied to obese patients, as sound transmission through the subcutaneous tissue cannot be excluded. Our studies showed that the signal of subjects with increased body mass index was of higher amplitude than in patients with normal weight. Age, however, does not appear to affect the signal, because a covariate analysis failed to detect age as a factor influencing the LFU signals. However, further studies to define the effect of age and gender are needed. It appears that the necessary degree of cooperation may be less than with conventional FEV1 measurements because meaningful measurements are possible with non-forced maximum expiration. Why the LFU method was even more sensitive with non-forced as opposed to forced expiration breathing is unclear at present; whether this might be explained by the speed and distortion of the chest wall displacement needs to be determined.

## Conclusions

In summary, we have shown that low frequency ultrasound is able to detect air trapping in COPD patients of GOLD stage 1-3 and appears to be a useful additional tool in the screening for COPD. It is inexpensive, easy to perform and noninvasive, so it could be applied during routine checks in general practice medicine to monitor air trapping. In contrast to spirometry and body plethysmography, no special training is required. We conclude that low frequency ultrasound might be helpful in identifying air trapping and in deciding which patients should undergo more specialized lung function testing. We suggest that further studies with more study subjects should be performed to define reference values and to further standardize the measuring procedure.

## Abbreviations

BMI: body mass index; COPD: chronic obstructive pulmonary disease; GOLD: the Global Initiative for Chronic Obstructive Lung Disease; LFU: low frequency ultrasound; R: airway resistance.

## Competing interests

The authors declare that they have no competing interests.

## Authors' contributions

The study was conceived and planned by KM, HB, TG, SU and JMH. The sensors and the software were made by FM, SL and DR. The study was conducted by KM and HB. KM performed the initial data analysis and HB wrote the draft of the manuscript. All authors participated in the interpretation of the data and the writing of the manuscript. All authors read and approved the final manuscript.

## Pre-publication history

The pre-publication history for this paper can be accessed here:

http://www.biomedcentral.com/1471-2466/12/8/prepub

## Supplementary Material

Additional file 1 **Supplementary figure. 1 Relationship between BMI and the inspiratory and the expiratory signals**. The high pass frequencies were determined for inspiration (circle) and the expiration (triangle). Linear regression analysis showed the following correlation between the BMI and the high pass frequencies: r^2 ^inspiration: 0.29 (p = 0.0046); r^2 ^expiration: 0.66 (p < 0.0001). Click here for file
