# Supplementary figures and images for: Detection of air trapping in chronic obstructive pulmonary disease by low frequency ultrasound
Source: BMC Pulm Med. 2012 Mar 16;12:8. doi: 10.1186/1471-2466-12-8 (PMC3359201; doi:10.1186/1471-2466-12-8)

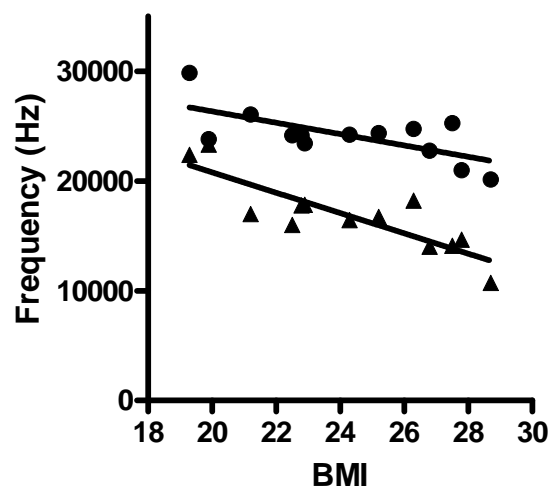

Suppl. Fig. 1

Supplement: Additional file 1 — Supplementary figure. 1 Relationship between BMI and the inspiratory and the expiratory signals. The high pass frequencies were determined for inspiration (circle) and the expiration (triangle). Linear regression analysis showed the following correlation between the BMI and the high pass frequencies: r2 inspiration: 0.29 (p = 0.0046); r2 expiration: 0.66 (p < 0.0001). [file 1471-2466-12-8-S1.PDF]
